# Supplementary material for: Causal Relationship between Aging and Anorexia Nervosa: A White-Matter-Microstructure-Mediated Mendelian Randomization Analysis
Source: Biomedicines. 2024 Aug 16;12(8):1874. doi: 10.3390/biomedicines12081874 (PMC11351342; doi:10.3390/biomedicines12081874)
Supplement: Supplementary file 1 [file biomedicines-12-01874-s001.zip › Supplementary Figure/Figure S4/Singe SNP plot of MR analysis for TL to ubm-b-1826.pdf]

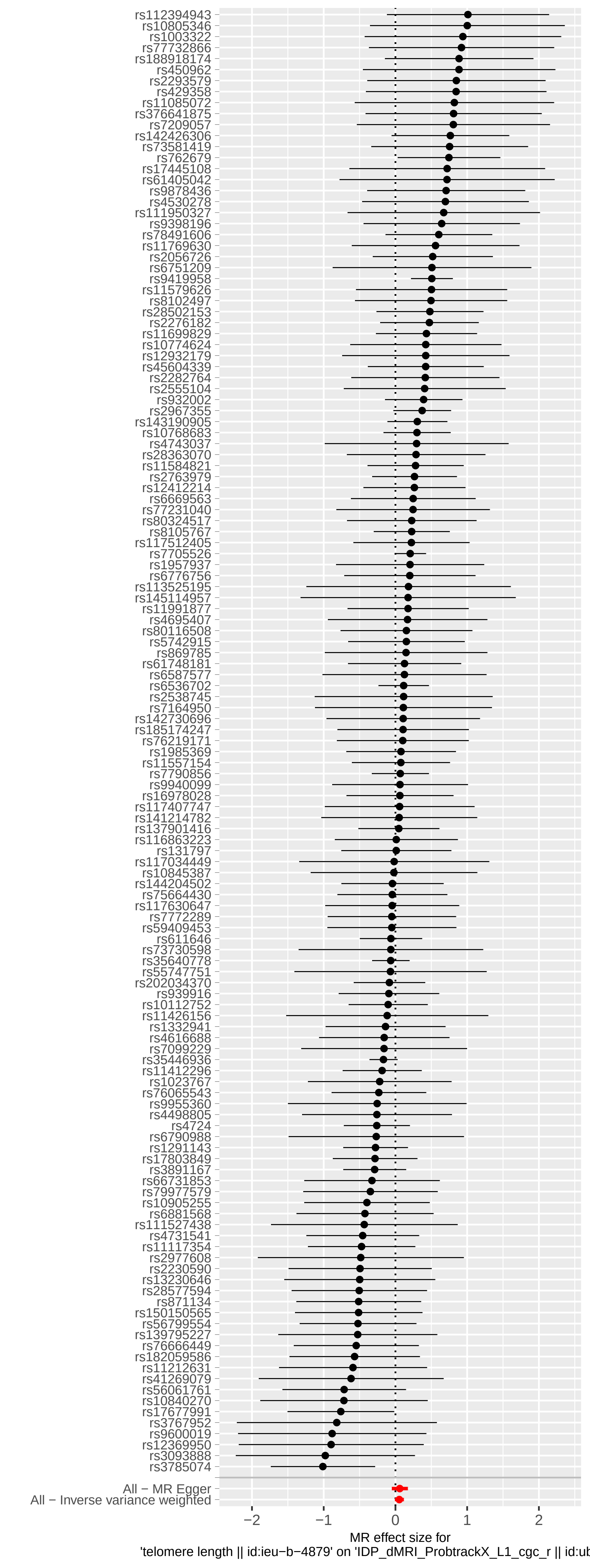
rs112394943
rs10805346
rs1003322
rs77732866
rs188918174
rs450962
rs2293579
rs429358
rs11085072
rs376641875
rs7209057
rs142426306
rs73581419
rs762679
rs17445108
rs61405042
rs9878436
rs4530278
rs111950327
rs9398196
rs78491606
rs11769630
rs2056726
rs6751209
rs9419958
rs11579626
rs8102497
rs28502153
rs2276182
rs11699829
rs10774624
rs12932179
rs45604339
rs2282764
rs2555104
rs932002
rs2967355
rs143190905
rs10768683
rs4743037
rs28363070
rs11584821
rs2763979
rs12412214
rs6669563
rs77231040
rs80324517
rs8105767
rs117512405
rs7705526
rs1957937
rs6776756
rs113525195
rs145114957
rs11991877
rs4695407
rs80116508
rs5742915
rs869785
rs61748181
rs6587577
rs6536702
rs2538745
rs7164950
rs142730696
rs185174247
rs76219171
rs1985369
rs11557154
rs7790856
rs9940099
rs16978028
rs117407747
rs141214782
rs137901416
rs116863223
rs131797
rs117034449
rs10845387
rs144204502
rs75664430
rs117630647
rs7772289
rs59409453
rs611646
rs73730598
rs35640778
rs55747751
rs202034370
rs939916
rs10112752
rs11426156
rs1332941
rs4616688
rs7099229
rs35446936
rs11412296
rs1023767
rs76065543
rs9955360
rs4498805
rs4724
rs6790988
rs1291143
rs17803849
rs3891167
rs66731853
rs79977579
rs10905255
rs6881568
rs111527438
rs4731541
rs11117354
rs2977608
rs2230590
rs13230646
rs28577594
rs871134
rs150150565
rs56799554
rs139795227
rs76666449
rs182059586
rs11212631
rs41269079
rs56061761
rs10840270
rs17677991
rs3767952
rs9600019
rs12369950
rs3093888
rs3785074
All − MR Egger
All − Inverse variance weighted
−2
−1
0
1
2
MR effect size for
'telomere length || id:ieu−b−4879' on 'IDP_dMRI_ProbtrackX_L1_cgc_r || id:ub
